# Lipoprotein Abnormalities in Chronic Kidney Disease and Renal Transplantation

**DOI:** 10.3390/life11040315

**Published:** 2021-04-05

**Authors:** Carlo Maria Barbagallo, Angelo Baldassare Cefalù, Antonina Giammanco, Davide Noto, Rosalia Caldarella, Marcello Ciaccio, Maurizio Rocco Averna, Emilio Nardi

**Affiliations:** 1 Department of Health Promotion, Mother and Child Care, Internal Medicine and Medical Specialties—University of Palermo, Via del Vespro, 127, 90127 Palermo, Italy; carlo.barbagallo@unipa.it (C.M.B.); abaldassare.cefalu@unipa.it (A.B.C.); agiamman@gmail.com (A.G.); notoddd@gmail.com (D.N.); liacaldarella@virgilio.it (R.C.); maurizio.averna@unipa.it (M.R.A.); 2 Department of Biomedicine, Neuroscience and Advanced Diagnostics (BIND), Section of Clinical Biochemistry, Clinical Molecular Medicine and Laboratory Medicine, University of Palermo, 90127 Palermo, Italy; marcello.ciaccio@unipa.it

**Keywords:** chronic kidney disease, lipids, lipoproteins, cardiovascular disease

## Abstract

Chronic kidney disease (CKD) is one of the most important risk factors for cardiovascular disease (CVD). Despite the kidney having no direct implications for lipoproteins metabolism, advanced CKD dyslipidemia is usually present in patients with CKD, and the frequent lipid and lipoprotein alterations occurring in these patients play a role of primary importance in the development of CVD. Although hypertriglyceridemia is the main disorder, a number of lipoprotein abnormalities occur in these patients. Different enzymes pathways and proteins involved in lipoprotein metabolism are impaired in CKD. In addition, treatment of uremia may modify the expression of lipoprotein pattern as well as determine acute changes. In renal transplantation recipients, the main lipid alteration is hypercholesterolemia, while hypertriglyceridemia is less pronounced. In this review we have analyzed lipid and lipoprotein disturbances in CKD and also their relationship with progression of renal disease. Hypolipidemic treatments may also change the natural history of CVD in CKD patients and may represent important strategies in the management of CKD patients.

## 1. Introduction

Chronic kidney disease (CKD) is one of the most important risk factors for cardiovascular disease (CVD) [1]. Several studies have established that cardiovascular (CV) mortality increases with decreasing glomerular filtration rate (GFR), and all recent guidelines have included patients with CKD among those who have a defined high or very high risk in relation to the value of GFR [2,3]. In patients with CKD, the prevalence of major CV risk factors such as diabetes mellitus and hypertension is particularly high; also, many of the so-called “emerging” risk factors (e.g., endothelial dysfunction, inflammation, oxidative stress, etc.) are present in patients with CKD and can contribute to negatively modulate cardiovascular risk [4]. However, the frequent lipid (cholesterol and triglycerides) and lipoprotein (chylomicrons; very low-density lipoprotein-VLDL; intermediate-density lipoprotein-IDL; low-density lipoprotein-LDL; and high-density lipoprotein-HDL) alterations occurring in these patients play a role of primary importance in this context [5].

## 2. Chronic Renal Failure (CRF)

Although the kidney has no direct implications for lipoproteins metabolism CRF dyslipidemia is usually present in patients with CRF [6]. This metabolic alteration is characterized by both quantitative and qualitative modifications of lipoproteins that significantly impact the development of accelerated atherosclerotic lesions of these subjects and probably influence the progression of the renal disease itself [7,8]. Main lipids and lipoprotein abnormalities in CRF are summarized in the Table 1 and Table 2. CRF patients exhibit high triglyceride levels, low high-density lipoprotein (HDL) levels, and normal or even low total cholesterol and low-density lipoprotein (LDL) cholesterol levels, but an atherogenic profile is hidden behind this spectrum of lipoprotein derangement [9]. This profile includes the increase in apolipoprotein (apo) B, lipoprotein (a) (Lp (a)), intermediate (IDL) and very low-density lipoproteins (VLDL), small and dense LDL and the reduction of HDL [10,11]. Furthermore, in patients with more severe CRF, LDL are often modified, leading to the increased formation of oxidized LDL [12].

### 2.1. Triglyceride Rich Lipoprotein (TRL) Particles and CKD

**Figure 1:**
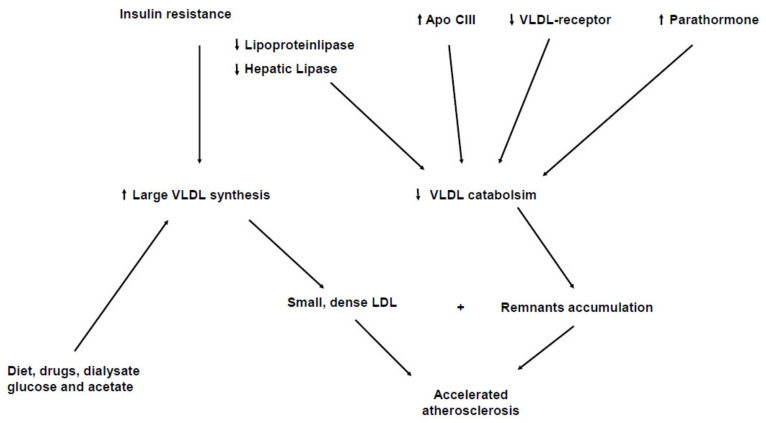
Mechanisms underlying hypertrigliceridemia in Chronic Kidney Disease.

Hypertriglyceridemia is a common feature in patients with CKD and this is due to an increased concentration of triglyceride-rich lipoproteins (TRL) (VLDL, chylomicrons and their remnants) [13,14,15]. Hypertriglyceridemia occurs both due to an increased production in the liver and to a delayed peripheral catabolism of TRL [16,17,18]. Figure 1 summarizes the main pathways involved on hypertriglyceridemia in Chronic Kidney Disease. An increased hepatic overproduction of VLDL may contribute to the increase in triglyceride levels in CKD patients [19,20]. Liver directly synthesizes VLDL, which, by the triglyceride hydrolase properties of lipoprotein (LPL) and hepatic lipase (HL), finally turns out to LDL [21]. LPL is an essential enzyme in the lipolysis of the triglyceride-rich lipoproteins (VLDL and chylomicrons), binds to the endothelial surface of the capillaries by interacting with the heparin sulfate proteoglycans and through the endothelium-derived glycosylphosphatidylinositol-anchored binding protein-1 (GPIHBP-1): this factor anchors LPL and acts as a ligand for chylomicrons [22]. LPL initially leads to the production of intermediated-sized lipoproteins (IDL and remnants), which may be either further processed by HL to form LDL or to be removed from plasma by receptor-mediated mechanisms [23]. Lipoproteins are actually heterogenous particles with discrete subspecies migrating in the same density interval and having a different atherogenetic role. IDL and remnants are rapidly removed from plasma in normal subjects, but represent potentially dangerous lipoprotein subpopulations [24]. Smaller and denser TRL particles are likely to better represent remnant lipoproteins as well as particles rich in apoE, and these specific TRL subspecies have been documented to be raised in CHD patients [25,26]. Enhanced hepatic lipoprotein synthesis and secretion should be induced by the hyperinsulinemia and insulin resistance often associated with CKD [27]. Insulin resistance activates the transport of free fatty acids (FFAs) to the liver and consequently promotes the oxidation or esterification of FFAs to cytosolic triglycerides or VLDL [28]. However the delayed catabolism seems to be the main mechanism responsible for the elevated concentration of TRL [29,30]. Despite secondary hyperparathyroidism playing a role in this impaired removal of TRL, this probably occurs due to a decrease in LPL and HL activities [31,32,33]. In addition, the presence of lipase inhibitors can further impair TRL catabolism [34]. CKD patients show higher levels of apolipoprotein CIII (apoC-III), which represent the physiological inhibitor of LPL [35,36]. It has been also demonstrated that VLDL of CRF patients is a poor substrate for bovine LPL in vitro [37]. Beyond low LPL and HL activities, these data suggest that structural alterations of LPL substrate might impair LPL activity in CKD [38,39], though CRF patients accumulate remnant particles and these abnormalities are commonly not detectable when fasting lipid profile is determined [40,41]. The causes underlying remnant accumulation in CKD patients are not completely understood. Although a down-regulation of hepatic receptors involved in IDL and remnant uptake may contribute to increase plasma residence time of these particles, impaired lipolysis might determine the accumulation of atherogenic remnants [42]. CKD patients have also a significant trigger of overproduction of TRL, which may potentially jeopardize the metabolism of remnant particles [43]. Combing both mechanisms, in a such defective lipolytic system, the remnant overloading could no longer permit an efficient removal of those particles, finally resulting in the accumulation of atherogenic TRL remnant fractions into the bloodstream. Since advanced CKD lipoprotein abnormalities mainly affect TRL, these are poorly modified by statins, having in the LDL the main target by decreasing hepatic cholesterol synthesis and enhancing apolipoprotein B (apoB) receptor expression [44,45].

### 2.2. Other Lipoprotein Abnormalities and CKD

Although LDL cholesterol is generally not elevated in CKD patients, a higher prevalence of small, dense LDL has been found [46]. These particles are more easily oxidized and penetrate more easily into the endothelial wall; for this reason they are more atherogenic, and thus, subjects who have higher, smaller and denser lipoproteins are at higher atherogenic risk [47]. In CKD is also observed a significant increase of plasma levels of Lp(a), which is also affected by the GFR [11,48,49]. Lp(a) levels are genetically determined and represent a strong risk factor for cardiovascular and cerebrovascular diseases [50]. Even if the mechanisms underlying the increase of Lp(a) in CKD are not completed clarified, it has been hypothesized that the kidney is involved in the catabolism of this particle [51]. In a homogeneous population of nondiabetic subjects without lipid-lowering therapy, serum proprotein convertase subtilisin kexin type 9 (PCSK9), which physiologically induces the degradation of LDL receptor, was not associated with GFR at several stages of CKD. However, PCSK9 was involved in the altered metabolism of TRL observed in CKD [52]. Thus, even if kidney function per se does not directly influence significantly PCSK9 metabolism, the real role of PCSK9 inhibitors in CKD should be considered. Another lipoprotein abnormality in CKD patients concerns HDL. The main function of HDL is the reverse transport of cholesterol, a process that includes the transport of cholesterol from the arterial wall to the liver for further excretion. Patients with CKD exhibit reduced HDL-cholesterol levels [53,54]. This is due to lower levels of apolipoprotein AI and AII, major components of HDL, but also to impaired activity of lecithin-cholesterol acyltransferase (LCAT), the key enzyme for the esterification of free cholesterol into HDL [55]. Moreover other processes involved in HDL metabolism are altered in CKD, such as an increased transfer of cholesterol esters to triglyceride-rich lipoproteins by the cholesterol ester transfer protein (CETP), a reduced activity of HDL-associated enzymes, such as paraoxonases, which may be responsible for the altered antioxidant and anti-inflammatory function of HDL [56,57]. All of these factors may contribute to reduce the antiatherosclerotic properties of HDL in this patient population.

A recent study has analyzed the association between the change of lipoprotein and the kidney outcomes in patients with stage 3–5 CKD over 3.2 years of follow-up. It has been shown that stage 3 CKD subjects with increased and high variable LDL-C levels exhibit an increased risk of progression to advanced CKD stages to dialysis. No significant difference on this correlation was found in patients with CKD stage 4 or 5. These results suggest that an efficacious lipid-lowering treatment is crucial to improve clinical outcomes, especially in patients with CKD stage 3 [58].

### 2.3. Lipid Abnormalities in CKD Patients on Hemodialysis and Peritoneal Dialysis

Patients with end-stage renal failure (ESRF) treated with different dialytic procedures (hemodialysis–HD–or continuous ambulatory peritoneal dialysis–CAPD) exhibit a different pattern of dyslipidemia. It was found that ESRF subjects on HD mainly present with hypertriglyceridemia, high apoB levels, low HDL-C and low apoA-I levels, while in patients on CAPD cholesterol serum levels also are increased [15]. Lp(a) levels are a 3-fold increase both on HD and CAPD compared to nonadvanced CKD patients [15]. Hemodialysis also determines acute relevant changes of lipoprotein profile, leading to a massive reduction of plasma triglyceride and increase of HDL-cholesterol level [59,60,61]. This is due to the acute lipolysis stimulation determined by heparin [62]. Heparin solubilizes LPL from the vascular endothelium and only the released enzyme exerts the metabolic effect on TRL [63]. It is not clear if these represent positive changes. LPL activity during heparin administration increases rapidly in ESRF subjects during the first hour, with a consequent triglyceride reduction, but still remains only half of that of normal controls [64]. In a previous work, we reported a selective decrease of the large triglyceride-rich particles with a concomitant raise of smaller VLDL and IDL together with apoE-rich fractions and non-HDL cholesterol levels [65]. Thus, at the end of dialysis procedure, there is an acute production of atherogenic TRL particles. CRF patients have to be treated by hemodialysis three to four times/week, so the adverse effect of the periodical heparin infusion should be accurately considered [66]. In EDRF patients treated by CAPD patients, an increased lipoprotein production appears to be the prevalent pathogenetic mechanism. In this category of subjects there are also higher levels of total and LDL cholesterol in comparison with other CKD patients [67,68]; the high absorption of glucose from the dialysis fluid probably lead to an increase in insulin levels resulting in the liver overproduction of lipoproteins [69]. In addition, acetate present in CAPD dialysis fluids might influence lipogenetic biochemical pathways and enhance VLDL secretion with a higher prevalence of hyperapobetalipoproteinemia [15]. HD and CAPD are usually similar in terms of long-term survival even though the risk factors for cardiometabolic syndrome are different between the two procedures. It has been demonstrated that patients on CAPD have a high prevalence of metabolic syndrome with weight gain and BMI increase, high fasting blood glucose, high HbA1c levels, insulin resistance and LDL-C increase, mainly due to extra calories from the dialysis fluid when compared to subjects on HD [70]. Recently it was shown that CKD patients on CAPD exhibit higher systolic and diastolic blood pressure than HD subjects regardless of slow and continuous manner of their procedure [71]. This is probably due to the hypervolemia condition consequent to high sodium intake, insufficient ultrafiltration or wrong evaluation of their dry weight [72]. On the contrary, Tonbul et al. reported that patients on HD present often with higher left ventricular mass index and are frequently nondippers compared with patients on CAPD [73]. Serum albumin is lower in CAPD patients due to several factors: among these, the significant loss of albumin during the procedure, the increased protein catabolic state and often the small protein intake typical of CKD patients [71]. Glucose-based dialysates exert systemic glucotoxic effects that may increase the risk of inflammation, oxidative stress and atherosclerosis [73]. In addition, on CAPD, plasma fibrinogen and serum homocysteine levels are significantly higher than on HD, even though there are not differences in subclinical atherosclerosis [70] as well as in hs-CRP and Lp(a) levels [74]. CRP indirectly promotes cardiovascular events through the activation of monocytes and complement factors, thus exposing patients on CAPD to high prevalence of major acute cardiovascular events compared with subjects on HD [71]. Further studies in this field are needed to elucidate if additional factors may be involved in the pathogenesis of cardiovascular disease in CKD patients regardless of the dialysis procedure applied.

## 3. Nephrotic Syndrome

Nephrotic syndrome is one of the most common manifestations of glomerular damage characterized by proteinuria (>3.5 g/1.73 m^2^/day), low albuminemia levels, edema and hyperlipidemia. The latter is the result of different modifications of lipoprotein metabolism in terms of both qualitative and quantitative changes [75,76], independently of any progression to CKD. Plasma cholesterol, triglycerides as well as all apo B-containing lipoproteins (VLDL, IDL, LDL and Lp(a)), are increased in nephrotic syndrome; on the contrary HDL are normal or decreased [75]. These disorders are mainly caused by alterations in the activity of the key factors that are candidates in the regulation of the physiological steps (assembly, transport, secretion, catabolism) of lipoproteins. Nephrotic syndrome is characterized by an important increase of serum total cholesterol and LDL cholesterol due to alterations of the mechanisms of production/clearance of LDL and apoB-100 [77,78]. PCSK9 and the liver tissue inducible degrader of the LDL receptor (IDOL) are increased in this specific kidney disease and together determine an important LDL receptor deficiency, a decreased hepatic uptake of LDL and consequently increased LDL cholesterol plasma levels [79]. In addition, nephrotic syndrome determines a significant increase of the activity of the acyl-CoA cholesterol acyltransferase-2 (ACAT-2) in the liver, thus inducing a growth of the cholesterol esterification, a decrease of the intracellular free cholesterol and the activation of 3-hydroxy-3-methylglutaryl-CoA (HMG-CoA) reductase, thus enhancing the cholesterol production and consequently hypercholesterolemia [80]. Several studies have shown a lack of LPL in nephrotic syndrome probably due to post-transcriptional or post-translational alterations involving also its cofactor GPIHBP1 [81]. LPL and GPIHBP1 downregulation are also associated with the apolipoprotein E and apolipoprotein CII decrease and apo CIII/ CII ratio increase in TRL [81]. Another intermediary in LPL deficiency is represented by ANGPTL4, a glycoprotein expressed in several tissues that physiologically inhibits LPL activity and is upregulated in nephrotic syndrome as emerging studies have demonstrated [82], thus contributing to hypertriglyceridemia pathogenesis. Consequently LPL-mediated lipolysis of VLDL and chylomicrons is inadequate and causes the progressive accumulation of serum triglycerides, the increase of VLDL triglycerides content, the impaired clearance of chylomicrons and the lipemia post-meal observed over nephrotic syndrome [76]. Hypertriglyceridemia is also related to HL and VLDL receptor deficiency. HL is involved in the IDL hydrolysis and their clearance of the TG content to be transformed in LDL. It has been shown that also in nephrotic syndrome the dysfunction of these factors induces an increase of atherogenic IDL and triglyceride accretion of LDL and HDL content [83,84]. Nephrotic syndrome determines several alterations in the morphology and functions of HDL, mainly due to impairment of their key structural proteins and of the reverse cholesterol transport process [85], thus influencing the atherogenic manifestations of this renal injury. Furthermore, Lp(a) is marked increased in nephrotic syndrome [86], mainly due to hypoalbuminemia through a process requiring apoB enhanced production, which determines an augmented synthesis of LDL particles to be combined into Lp(a) [87].

## 4. Renal Transplantation

Renal transplantation is usually characterized by a whole variation of lipid profile [88]. Triglyceride levels decrease, while total and HDL-cholesterol levels significantly increase [88]. It has been shown there is an enhanced production of VLDL by the liver associated with a normal conversion to LDL since LPL activity comes back to normal [88]. On the other hand, lipoprotein abnormalities in renal transplant recipients (RTR) look to depend essentially to the considerable pharmacological interventions to which these patients are exposed; they do not regress automatically and they may affect a large number of subjects over time [89]. In RTR, the main lipid alteration is hypercholesterolemia [87,90], while hypertriglyceridemia is less pronounced. HDL cholesterol levels has been found normal or high in this category of subjects; HDL subfractions are variable since some researchers have demonstrated low levels of HDL2 [91] while others failed to show any decrease of HDL2 particles [92]. Lp(a) are only slightly increased when kidney function improves [15], but its metabolism is not completely known. ApoA-I and apoB are significantly high, and apoC-II, apoC-III and apoE are significantly low. These observations suggest an increased cardiovascular risk in these subjects, and the increase of HDL-C and apoA-I might not be a protective factor. On the other hand, it has been shown that a Mediterranean diet may benefit by inducing an inversion of the increased trend of total cholesterol levels [93]. Recently, a study by Kim JE et al. analyzed the ratio of triglyceride to HDL-C and major cardiovascular events (MACE) in RTR, and showed a significant correlation: based on their findings, the maintenance of acceptable TG/HDL-C levels in these patients may decrease CV risk and increase long-term graft survival [94]. Further studies are needed to clarify the correlation between the lipid abnormalities described and the risk for cardiovascular diseases.

## 5. Hypolipidemic Treatments and CKD

Three large randomized clinical trials have been specifically carried out in patients with CKD to assess the effect of lipid-lowering treatment with statins on clinical outcomes: Die Deutsche Diabetes Dialyse Studie (4D), A Study to Evaluate the Use of Rosuvastatin in Subjects on Regular Haemodialysis: An Assessment of Survival and Cardiovascular Events (AURORA) and Study of Heart and Renal Protection (SHARP) [95,96,97] (see Table 3). While 4D and AURORA included only patients on hemodialysis, the SHARP trial enrolled patients with CKD at different stages. 4D and AURORA, despite a significant reduction of LDL-cholesterol by atorvastatin and rosuvastatin, respectively, failed to demonstrate effects on major CV events. In the SHARP trial, treatment with simvastatin/ezetimibe reduced the rate of major atherosclerotic events in all subjects except those on hemodialysis at the time of study inclusion [97]. Thus, lowering of LDL-C with statins appears to reduce atherosclerotic CV events in CKD patients who are not in hemodialysis. A more recent meta-analysis confirmed that the rate of reduction of CV events becomes smaller when GFR declines [98]. It is possible that in hemodialysis, atherosclerotic patients are so advanced to eliminate the effectiveness of any lipid-lowering intervention. The ESC/EAS guidelines have taken into account this information, recommending treatment with statins/ezetimibe in patients with CKD still in conservative therapy [2]. The study ALERT (Assessment of Lescol in Renal Transplantation) showed the significant effect of fluvastatin in preventing and decreasing the risk for CV events in kidney transplant recipients [99].

Moreover, lipid management in CKD subjects is based on recommendations from the major clinical practice guidelines as KDIGO and ACC/AHA, which suggest the use of statins rather than other lipid-lowering therapies, since their efficacy has been already tested in randomized controlled trials (RCTs) [100]. According to the KDIGO guideline, subjects with CKD stages 3–5 (not on dialysis) as well as patients with CKD stages 1–2 (eGFR > 60 mL/min/1.73 m^2^) aged ≥50 years who have pathological albuminuria (urinary albumin:creatinine ratio >30 mg/g) should be treated with a statin or statin plus ezetimibe combination [99].

KDIGO recommends treatment of dyslipidemia on the basis of CHD risk, which depends maximally by age. However this guideline does not have an upper age limit for therapy recommendations contrary to ACC/AHA guidelines that suggest avoiding statin therapy for primary prevention in CKD patients older than 79. Moreover, KDIGO suggests a statin dose decrease in subjects with an eGFR < 60 mL/min/1.73 m^2^. So far, there are not randomized trials indicating the benefits of statins in patients with nephrotic syndrome. The effect of statin treatment on mortality in CKD subjects depends on the CKD stage. Patients with stages 2–4 CKD on statin therapy, do not exhibit any difference in terms of mortality compared to placebo. However it has been shown that statins can reduce the mortality in stage 5 CKD subjects [101].

Subgroups of FOURIER and ODYSSEY Outcomes trials have also demonstrated a similar benefit of PCSK9 inhibition independent of kidney function, despite subjects with severely reduced kidney function being excluded [54,102,103], confirming that PCSK9 inhibition might represent an alternative treatment approach in patients with moderate CKD.

There are limited data on PCSK9 inhibitors in CKD. The FOURIER trial evaluated the efficacy and safety of Evolocumab in CKD patients, and a recent analysis of 8 randomized trials on Alirocumab reported that these agents decrease LDL-C and reduce the CV risk among subjects with mild to moderate CKD spectrum. However these studies have some limitations that reflect the small number of patients with stage 3b and 4 CKD and the exclusion of patients with eGFR < 20 mL/min/1.73 m^2^. Furthermore, their benefits are dependent of the degree of cholesterol lowering and it seems that their efficacy decreases as CKD turns into more severe stages. Further studies are needed to prove the suitability of PCSK9 inhibitors in the treatment of CKD patients [99].

Other lipid-lowering agents are fibrates but to date, very little proofs recommend their use in subjects with CKD, especially in those with eGFR < 30 mL/min//1.73 m^2^. The only exception might be very high triglyceride levels (>11.3 mmol/L (>1,000 mg/dL) in which these drugs should be used reasonably by adjusting the dose according to kidney function [99].

Bile acid sequestrants such as cholestyramine, colestipol and colesevelam are actually administered as second-line options in subjects with atherosclerotic CV disease, but there is only a little evidence for their use in CKD population. Moreover, since these agents might increase triglyceride levels, their utility in subjects with CKD or ESRF is very limited. To date, there is poor evidence to support the use of omega-3 fatty acids to decrease CV outcomes or ameliorate mortality in patients with CKD or ESRF. On the other hand, two placebo-controlled RCTs, REDUCE-IT (Reduction of Cardiovascular Events with EPA-Intervention Trial) and STRENGTH (Outcomes Study to Assess Statin Residual Risk Reduction with Epanove in High CV Risk Patients with Hypertriglyceridemia), are evaluating the effect of high-dose (4 g) eicosapentaenoic acid on CV outcomes in subjects with hypertriglyceridaemia [99].

## 6. Lipoprotein Abnormalities and Progression of CKD

Taken together, all these events contribute to the development of cardiovascular diseases (i.e., atherosclerosis) and, at the same time, to the progression of kidney disease [76]. Indeed, more than 30 years ago, the “lipid nephrotoxicity hypothesis” was proposed, in which dyslipidemia may increase the risk of atherothrombotic events, nephrotoxicity, glomerulosclerosis and progressive kidney disease [104]. Hyperlipidemia, particularly TRL and oxidized LDL, induces the production of cytotoxic metabolites, cytokines and reactive oxygen species (ROS) by mesangial cells, causing an injury of the glomerular epithelial and endothelial cells, thus resulting in glomerulosclerosis [105]. It was observed that, in nephrotic syndrome, the free fatty acids are increased and bind to albumin, causing podocyte damage with consequent loss of their morphology and tubulointerstitial injury [104], but the role of dyslipidemia in the pathophysiology of the nephrotic syndrome is still poorly understood [104]. In animal models, lipid alterations induce glomerular and tubular damage, with positive effects induced by hypolipidemic therapy [105,106,107,108]. The mechanisms are not fully understood, but the main hypothesized explanation is related to the inhibition of mevalonate, a known stimulant of cell replication and glomerular proliferation [109]. This accounts for the potential beneficial effect of statins. In humans, association between high LDL levels and decline in renal function in dyslipidemic patients was reported [110]. Conversely, others have shown that high triglyceride levels and low HDL levels are independent risk factors for renal dysfunction but LDL cholesterol levels were not predictive of kidney damage in a relatively short time [111]. Over a longer follow-up period, a significant association between abnormal lipoprotein parameters and the development of renal dysfunction was shown [112]. Moreover, the effects of hypolipidemic treatment on renal disease progression are controversial. In particular, only high-intensity statin therapy seems to improve GFR decline, while low- and moderate-intensity statins did not achieve the same positive results, and statin therapy did not reduce, or even increase, proteinuria in CKD patients [113,114,115,116].

## 7. Conclusions

Metabolism of lipids and lipoproteins is substantially altered in CKD and the frequent alterations occurring in these patients may promote atherogenesis, playing a role of primary importance in the development of CVD. Elevated plasma levels of apoC-III, apoA-I and the apoB-containing lipoproteins may modify lipoprotein metabolism, leading to the accumulation of atherogenic particles and a profile characterized by elevated triglycerides, Lp(a), dense LDL and low HDL cholesterol. Different enzymes pathways and proteins involved in lipoprotein metabolism are impaired in CKD, and these changes are mainly dependent of the different setting of kidney disease as well as the kidney transplant recipient state. These modifications may be controlled and, although there are limited data on CKD population, lipid-lowering therapies including statin or statin plus ezetimibe combination, PCSK9 inhibitors, omega 3 and fibrates (in selected cases and based on eGFR value) represent important strategies in the management of CKD patients and in the prevention of CV events.

## Figures and Tables

**Table 1:** Lipids and lipoprotein abnormalities in CKD patients.

↑ Triglycerides
↑ TG-rich particles
= to ↑ LDL-cholesterol
↓ HDL-cholesterol
↑ Non HDL-cholesterol
↑ Small, dense LDL
↑ Remnants
↑ Lipoprotein(a)
↑ Modified LDL
↑ Apo CIII (in VLDL)
↓ Apo AI/AII
↓ LPL
↓ HL
↓ L-CAT
↑ CETP
= PCSK9
↓ Paraoxonase

CKD: Chronic Kidney Disease; TG: Triglyceride; LDL: Low-density lipoprotein; HDL: High-density lipoprotein; Apo CIII: Apolipoprotein CIII; VLDL: Very low density lipoprotein; Apo AI: Apolipoprotein AI; Apo AII: Apolipoprotein AII; LPL: Lipoprotein lipase; HL: Hepatic lipase; L-CAT: Lecitin-Cholesterol Acyl Transferase; CETP: Cholesteryl ester transfer protein; PCSK9: Proprotein Convertase Subtilisin/Kexin type 9.

**Table 2:** Comparison in the main lipid and lipoprotein abnormalities between different types of CKD.

Lipoproteins	Nephrotic Syndrome	Non-Nephrotic CKD	Diabetic CKD	HD	CAPD	Kidney Transplantation
**TC**	↑↑	↓	↑	↑↑	↑↑	↑↑
**HDL-C**	Normal or ↓	↓	↓	↓↓	↓↓	Normal or ↑↑
**LDL-C**	↑↑	↓	↑			↑↑
**Triglycerides**	↑↑	↑↑	↑↑	↑↑	↑↑	↓↓
**VLDL**	↑↑	↑↑	↑↑	↑↑	↑↑	↓↓
**Chylomicrons**	↑↑	↑↑	↑↑	↑↑	↑↑	↓↓
**IDL**	↑↑	↑↑	↑	↑	↑	↓↓
**Lipoprotein(a)**	↑↑	↑↑	↑	↑	↑	↑
**ApoB**	↑↑	↑↑	↑	↑↑	↑↑	↑↑
**Apo-A1**	Normal or ↓	↓	↓	↓↓	↓↓	↑↑

CKD = Chronic Kidney Disease; HD = Hemodialysis; CAPD = continuos ambulatory peritoneal dialysis; TC = Total cholesterol; HDL-C = HDL-cholesterol; LDL-C = LDL-cholesterol; VLDL = Very-low-density lipoprotein; IDL = Intermediate-density lipoprotein; ApoB = Apolipoprotein B; Apo-A1 = Apolipoprotein A1.

**Table 3:** Trials evaluating the reduction of cholesterol-LDL and cardiovascular risk in specific CKD populations.

Trial	Population	No. Patients	Drug	Follow-Up (Years)	Ldl Reduction %	Cv Event Reduction %	*p* Value
SHARP	CKD patients	9270	Simvastatin 20 mg/ ezetimibe	4.9	55%	17%	0.0021
4D	Hemodialysis	1255	Atorvastatin 20 mg	4	42%	-	0.35
AURORA	Hemodialysis	2776	Rosuvastatin 10 mg	3.8	43%	-	0.59

LDL: Low-density lipoprotein; CKD: Chronic Kidney Disease; CV: Cardiovascular; SHARP: Study of Heart and Renal Protection; 4D: Die Deutsche Diabetes Dialyse Studie; AURORA: An Assessment of Survival and Cardiovascular Events.

## Data Availability

Not applicable.
